# Supplementary figures and images for: The effect of the sugar metabolism on Leishmania infantum promastigotes inside the gut of Lutzomyia longipalpis: A sweet relationship?
Source: PLoS Negl Trop Dis. 2022 Apr 6;16(4):e0010293. doi: 10.1371/journal.pntd.0010293 (PMC8985994; doi:10.1371/journal.pntd.0010293)

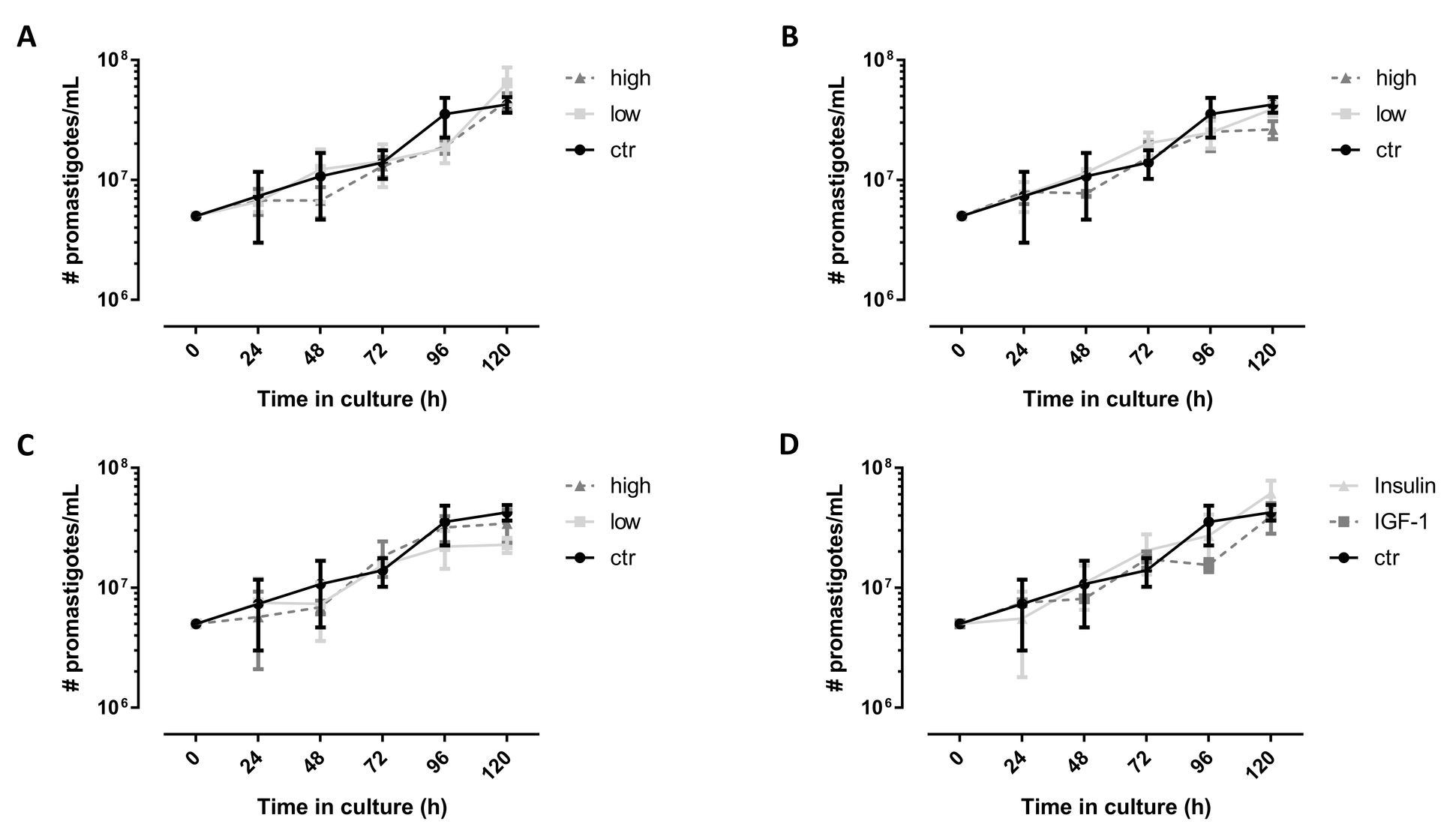

Supplement: S1 Fig — Comparison of in vitro promastigote growth of L. infantum cultured in either medium without additives (ctr) or in media containing low of high levels of glucose (A), fructose (B) or galactose (C) or insulin and IGF-1 (D). No significant differences in L. infantum promastigote growth could be observed. The parasite density at each time point of cultivation is the average of at least two independent experiments run in duplicate ± the standard error of the mean. (TIF) [file pntd.0010293.s001.tif]
