# Supplementary material for: The effect of the sugar metabolism on Leishmania infantum promastigotes inside the gut of Lutzomyia longipalpis: A sweet relationship?
Source: PLoS Negl Trop Dis. 2022 Apr 6;16(4):e0010293. doi: 10.1371/journal.pntd.0010293 (PMC8985994; doi:10.1371/journal.pntd.0010293)
Supplement: Attachment — Submitted filename: Answer to the reviewers final.docx [file pntd.0010293.s002.docx]

**Answer to the reviewers**

**Editor:**

1. Figure 1: the figure lacks the panels D and E that are advertised in the text. Moreover, the results shown in the figure (panel B) do not match your description in the text; the R-square values differ. Lastly, the Figure legend is insufficient and must be amended with all pertinent information needed for understanding your experimental set-up, including sample sizes and number of biological repeats.

The authors apologize for the error that has slipped into figure 1 during the various rounds of internal revision by the authors. Figure 1 has been adjusted, now containing panels A-E. The figure legend has been adjusted and contains the necessary information on sample size and biological repeats. The r-values mentioned in the text have been carefully revised and the R² values shown in the graphs have been changed into the r-values discussed in the text to avoid further confusion.

2. Figure 2 does not convey important information and should be shifted to the supplementary information.

The authors have placed figure 2 into the supplementary material, as suggested.

3. For Figures 3ff, state how many repeat experiments were conducted. The figures only display the number of sandflies that were evaluated. Likewise, please state what measures, e.g. double-blind counting, were taken to avoid observational bias since you relied heavily on microscopic evaluations.

As stated in the figure legend two repeat experiments were performed. This information, together with the measures for randomization to avoid bias were added in the materials and methods section.

3. line 167: "In parallel, an estimated percentage of metacyclics was calculated based on parasite morphology and motility. " Not sufficient. What were the parameters used to distinguish between procyclics and metacyclics and where were those parameters first described and/or evaluated (Citations)?

The percentage of metacyclic parasites within the sand fly gut was based on microscopic examination of differential morphological and motility characteristics between metacyclics and procyclics. The microscopist has ample experience in differentiating the metacyclic and procyclic parasites in a KOVA counting chamber. This information and appropriate references have been added to the manuscript.

4. Please make clear, that the majority of the observed effects are transient, in the text, the conclusions and the abstract.

The authors have highlighted in all sections of the manuscript that the recorded effects were indeed transient although especially the earlier appearance of metacyclic parasites and moderate differences in survival deserve proper highlight in the various sections.

***Methods***

**Reviewer #1**: The study has a clear hypothesis that was tested using appropriate methods, but some information should be added.

I miss the information if infected sand flies were further provided with a 30% glucose solution as the colony. This is important as this additional sugar supply may decrease or fully cover between-group differences of glucose intake in the blood.

This information has been added in the manuscript. The flies were indeed fed on a 30% sucrose solution during the course of the experiment. The authors only wanted to focus on evaluating the impact of the host blood biochemistry on subsequent parasite development in the vector. As sand flies in nature would also feed on sugary plant secretions after having a blood meal, our experimental set-up was aimed to resemble this natural situation.

Also, number of sand fly females should be given for each group in Figs. 5 and 7 as this parameter is more important than the number of observations.

The number of observations is the same as the number of female sand flies. This has been clarified in the manuscript as also suggested by reviewer 2.

**Reviewer #2:**

-Were correct statistical analysis used to support conclusions?

Partly, there are some mistakes in the figures regarding the analysis of statistical significance

The authors carefully checked all figures and all statistical analyses and made the necessary adjustments in the manuscript.

***Results***

**Reviewer #1:** The results are clearly described with exception of the first chapter “Correlation of host blood glucose levels with parasite burdens” (lines 180-185). Here, the text including statistical values does not correspond to Figure 1 and its legend. According to the figure, the correlation was found in the spleen only while in the text, correlation is declared also for the liver. This must be clarified as the statement that the correlation was significant also for the liver repeats in many parts of the manuscript. Also, Figure 1 has only three parts (A, B and C) while in the text, A-E parts are listed. In addition, two kinds of evaluation of parasite load are mentioned in the text but only one is described in Materials and Methods.

The authors apologize for the confusion. An error has indeed slipped into figure 1 during the various rounds of internal revision by the authors. Figure 1 has been adjusted, now containing panels A-E and all statistical analyses have been checked and both figure and text are edited accordingly.

**Reviewer #2:**

-Are the results clearly and completely presented?

No, Figure 1 is incomplete (E+D are missing)

Figure 1 has been corrected.

-Are the figures (Tables, Images) of sufficient quality for clarity?

I would recommend to improve all figures.

Figure 1 is incomplete

All figures and figure legends have been edited according to the reviewer’s comments.

***Conclusions***

**Reviewer #1**: Conclusions are not well supported by data and authors overvalue their findings of the effect of sugars on Leishmania development in sand flies: In Fig. 3 the little effect was demonstrated for medium glucose concentration on days 5 and 7 but not on late infection on day 9 (and no effects were demonstrated for fructose and galactose). In Fig. 4 little effect of glucose was demonstrated for metacyclogenesis as slight differences were seen on day 5 but not on days 7 and 9 (and the negative effect was found for other monosaccharides).

The authors have made several modifications to the manuscript to indicate that some of the observed effects are transient, and to shed a more nuanced light on their findings.

**Reviewer #2:**

-Are the limitations of analysis clearly described?

No. It is necessary to make limitations clear, especially the comparing to other insects/parasites/bacteria must be improved

The authors have elaborated on the experimental limitations in the discussion.

-Do the authors discuss how these data can be helpful to advance our understanding of the topic under study?

Yes, but minor improvements would be helpful

The authors have expanded on this in the discussion

-Is public health relevance addressed?

Too less, I would recommend to improve this.

The authors have expanded on public health relevance in the discussion.

***Editorial*** ***and*** ***Data*** ***Presentation*** ***Modifications***

**Reviewer #1:**

Abstract:

Line 26 – “ and because glycemic levels…..this sentence should be separated as it represents the result of this manuscript, not previously published facts like the beginning of the compound sentence does.

The sentence has been split into two.

Line 28 – “and hepatic” should be deleted as hepatic parasite burdens are not inversely correlated to glycemic levels according to data presented in Figure 1.

The reviewer is correct. The liver has been deleted from the sentence.

The last two sentences of the Abstract should be changed to better correspond to the described results: Higher carbohydrate and insulin/IGF-1 levels favored sand fly survival while all the recorded effects on parasite loads and appearance of metacyclics were modest, temporary and not apparent in late infections. Therefore, these observations do not support the concept that host blood biochemistry can directly affect Leishmania transmission.

Similarly, also Author Summary and the end of the Discussion should be rewritten.

The authors have carefully revised the manuscript and have modified abstract, author summary, discussion and conclusion to be more in line with the transient nature of the observed differences.

Figures 2, 5 and 7: in the current black and white presentation, lines for low and medium levels are not clearly distinguishable. I suggest using color presentation or patterned lines.

The graphs have been adapted according to the reviewer’s suggestions.

Lines 337-339: Results obtained with L. longipalpis –derived cell lines should not be interpreted as confirmation of the role of toll and lmd pathways in innate responsiveness of the sand fly against Leishmania. Instead, recent studies with L. longipalpis will be more appropriate for such discussion.

The authors agree with the reviewer and have added references to recent studies performed with *L. longipalpis* in the discussion.

Discussion is relatively long, in several aspects similar to Introduction and not focused to own results.

The authors have tried to shorten the discussion based on the comments of reviewer 1 and 2.

***Summary*** ***and*** ***General*** ***Comments***

**Reviewer #1:** The topic of the manuscript is actual; the authors focused on the correlation of host glucose metabolism with leishmania development in phlebotomine sand flies and fly survival. The results support the effect of Leishmania infection on glucose level in the host and the effect of blood sugars and insulin/IGF1 levels on sand fly longevity. On the other hand, effects of blood sugars and insulin/IGF1 levels on parasite loads and appearance of metacyclics were none or too modest, but always temporary and, more importantly, they were not apparent in late infections on day 9.

However, the “negative results” are also important and should be published with a proper interpretation.

I suggest major revisions and:

-	Clarify better the results of experiments on the correlation of host blood glucose levels with parasite burdens

-	Change interpretation of data concerned on the correlation of blood biochemistry with parasite development in sand flies (my suggestions for changing the Abstract are written above).

Based on the reviewer’s suggestions, the authors have made several changes throughout the manuscript.

**Reviewer #2:**

Major comments

The authors present an interesting study about the impact of host sugar metabolism, i.e. blood composition, for sandflies and Leishmania parasites. The performed experiments are reasonable and adequately to prove their message.

- The introduction should be more organized. The examples for the influence of glucose levels on parasites in general switches between different insects and hosts without mentioning this and the possible differences (ecological behavior etc.).

The authors have re-written the introduction to make the discrimination between different pathogens and insects more clear.

- The Introduction of Leishmania parasites in the beginning is too short

The authors have added a small general section on leishmaniasis in the introduction.

- Introduction: The comparison of increasing parasite growth/proliferation due to low glucose levels with increasing transmission due to high glucose levels is not correct. Please compare either the growth/proliferation on different glucose levels or the transmission.

This has been modified in the manuscript

- Most figures must be improved

The authors have adjusted all figures and figure legends according to the reviewer’s suggestions.

- Figure 1 is not complete, 1 D and 1 E are missing

Figure 1 has been completed.

- Discussion: It is important to differentiate between the different insects and infections (different parasites, bacteria), this is done too generalized. For example, Line 319-320: reference 43 is about the immune stimulation in Drosophila due to Vibrio cholerae infections. The immune response of insects to gram-negative bacteria and parasites can utilizes different pathways. This should be mentioned in the text.

The discussion has been carefully re-read and sections on different pathogens and insects are re-written.

Minor comments

The authors want to thank the reviewer for his thorough revision. All the suggested changes were made and the requested information was added in the manuscript. The authors chose to keep the current units of glucose, fructose, galactose, IGF-1 and insulin as these are in line with what can be found as reference in literature.
